# Evaluation of Cyanobacterial Bloom from Lake Taihu as a Protein Substitute in Fish Diet—A Case Study on Tilapia

**DOI:** 10.3390/toxins13100735

**Published:** 2021-10-19

**Authors:** Yan Huo, Yuanze Li, Wei Guo, Jin Liu, Cuiping Yang, Lin Li, Haokun Liu, Lirong Song

**Affiliations:** 1 State Key Laboratory of Freshwater Ecology and Biotechnology, Institute of Hydrobiology, Chinese Academy of Sciences, Wuhan 430072, China; huoyanzky@163.com (Y.H.); 15770080434@163.com (Y.L.); guowei2021080@ctbu.edu.cn (W.G.); liujin@ihb.ac.cn (J.L.); m18856183491_1@163.com (C.Y.); lilin@ihb.ac.cn (L.L.); 2 College of Life Sciences, University of Chinese Academy of Sciences, Beijing 100049, China

**Keywords:** low toxic cyanobacterial bloom, tilapia diet, feasibility, risk assessment

## Abstract

The utility of cyanobacterial bloom is often hindered by concerns about the toxin content. Over three years of investigation, we found that the toxin content of cyanobacterial bloom in Lake Taihu was always low in June and higher in late summer and autumn. The findings enabled us to compare the effects of diets containing low and high toxic cyanobacterial blooms on the growth and consumption safety of tilapia. There were no negative effects on the growth of tilapia, and the muscle seemed to be safe for human consumption in the treatment of 18.5% low toxic cyanobacterial bloom. Therefore, limitations of the utilization of cyanobacterial biomass can be overcome by selecting low toxic cyanobacterial bloom that can be found and collected in large lakes.

## 1. Introduction

Cyanobacterial bloom has become a critical issue due to its impact on aquatic wildlife and human health [1,2,3]. One of the most widespread bloom-forming cyanobacteria in freshwater lakes is *Microcystis* [4,5]. A review documented that as of 2016, *Microcystis* had been recorded in at least 108 countries [6]. Therefore, efforts have been made throughout the world to control, prevent, and mitigate cyanobacterial bloom, and cost-effective methods have been developed to reduce the risks [7,8,9,10]. For example, mechanical collection of cyanobacterial bloom is the most direct and effective strategy [11,12]. This method has been used as an emergency measure in several Chinese freshwater lakes, reducing the biomass of cyanobacterial bloom and removing nutrients such as nitrogen and phosphorus from the water [13,14]. In Lake Taihu, for example, more than 10 million tons (wet weight) of cyanobacterial biomass have been collected since 2007 [15]. Traditional treatment techniques for the mechanically harvested algal sludge include landfills and incineration; these methods not only waste energy but also pose secondary pollution [16]. Therefore, it is urgent to find an efficient, harmless, and low-cost way to deal with cyanobacterial bloom biomass.

Among many approaches trying to make use of cyanobacterial bloom biomass [17,18], a substitute of the biomass as aquafeed has been studied for decades due to its high protein content of approximately 50% [19,20,21]. The criteria of the feasibility of using cyanobacterial bloom for aquafeed generally include growth performance, consumer safety, and the amount of added cyanobacterial bloom biomass. For example, after exposure to diets containing 0.5–1.1% *Microcystis* bloom, the average body weight, total length, specific growth rate, and survival of threadfin shad were not significantly different from the control group [22]. Acuna et al. (2012b) also showed that there were no significant differences in body weight, total length, or survival of Sacramento splittail fed with *Microcystis* bloom (0.6–2.3%) compared to the controls [23]. Zhao et al. (2006a) reported that dietary *Microcystis* biomass of 1–5% in the feed had no negative effects on the feed conversion efficiency or survival of the Nile tilapia [24]. At higher levels of *Microcystis* biomass, however, fish were found to be less tolerant and had a greater accumulation of toxin [6,25]. A previous study reported that the body weight and specific growth rate of yellow catfish decreased significantly after feeding 18.4% *Microcystis* bloom [26]. The addition of 30% of *Microcystis* biomass to the diet inhibited the growth of goldfish [19]. Dong et al. (2009) indicated that the body weight, specific growth rate and feed conversion efficiency of hybrid tilapia fed with a *Microcystis* bloom diet (43.6%) were significantly lower than those of the controls [27]. Moreover, the accumulation of microcystin (MC) in muscle tissue of yellow catfish [26], goldfish [19], and hybrid tilapia [27] exceeded the tolerable daily intake (TDI) recommended by the World Health Organization (WHO), indicating that the fish were not safe for human consumption.

Based on growth and safety testing, most studies have suggested that fish can utilize a small amount of cyanobacterial bloom biomass (usually less than 5% in feed). The factor that limits the amount of added cyanobacterial biomass may be its higher toxin content [19,22,24,26,27,28,29]. These results have given rise to the perception that cyanobacterial bloom can hardly be utilized in large quantities as a protein substitute, which has led to a decline in research interest in this field. This arguably raises several questions, e.g., is it possible to find a stable source of cyanobacterial biomass with low toxin content? If so, can the biomass be utilized in large quantities as a protein substitute?

*Microcystis*, the major bloom-forming genus, contains both toxic and non-toxic species. During the blooming period, either toxic or non-toxic species can dominate solely, or co-exist. In the case of Lake Taihu, for example, the early bloom is usually composed of *Microcystis flos-aquae* and can last for one month or so [30]. On further examination, strains of *M. flos-aquae* produced no toxin or trace amounts of toxin (0–26 µg/g DW) [30], suggesting that the biomass can potentially be used for aquafeed in large quantities. Other studies have indicated that there were high proportions of low or non-toxic *Microcystis* during the blooming in Lake Oneida [31], Lake Mikata [32], and Lake Dianchi [33].

Tilapia is one of the most economically important freshwater fish species, and it is on its way to becoming a major supplier of protein both in the developed and the developing world [34]. Aquaculture is the main driving force behind the world production of tilapia, which has increased rapidly, from less than 1.19 million tonnes in 2000 to nearly 6.19 million tonnes in 2019 [35]. The Nile tilapia *(Oreochromis niloticus)* is the predominant cultured fish species worldwide. With a natural herbivorous/omnivorous feeding habit, the Nile tilapia can adapt to feed containing a high content of plant protein, including algae [36].

Based on previous studies and our analysis, we suggest that a large amount of low toxic cyanobacterial bloom biomass may be available. The present research aimed to answer the question of whether it is feasible to add large amounts of cyanobacterial bloom biomass with low toxins to aquafeed. Therefore, this study compared the effects of cyanobacterial bloom biomass with high or low toxin content on the growth of tilapia and assessed the safety of the tilapia for human consumption. Our findings are not only beneficial to the aquafeed industry but also relevant to the utilization of cyanobacterial bloom biomass.

## 2. Results

### 2.1. The Effects of Dietary Cyanobacteria on Growth of Tilapia

**Figure 1:**
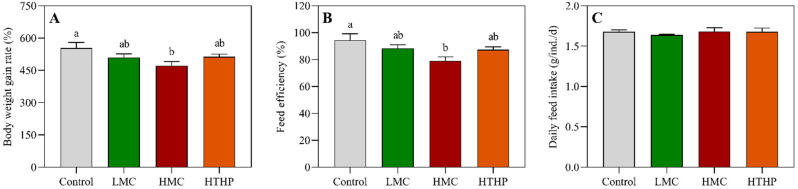
Effects of dietary cyanobacteria on growth performance and feed conversion of tilapia. (**A**) Body weight gain rate of the fish under different treatments; (**B**) Feed efficiency of the fish under different treatments; (**C**) Daily feed intake of the fish under different treatments. Different superscripts indicate significant difference (*p* < 0.05). Control: commercial diet; LMC: low microcystin content diet; HMC: high microcystin content diet; HTHP: high temperature and high press treatment.

**Figure 2:**
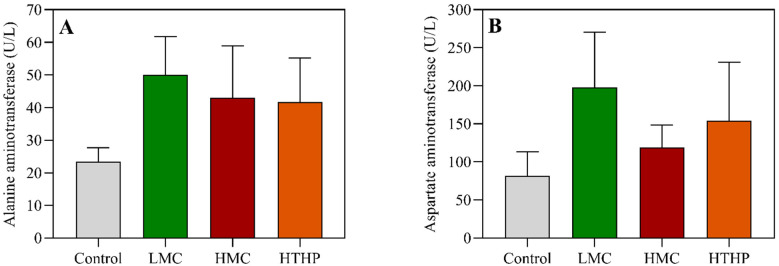
Effects of dietary cyanobacteria on ALT and AST activity in tilapia. (**A**) The ALT activity of the fish under different treatments; (**B**) The AST activity of the fish under different treatments. Control: commercial diet; LMC: low microcystin content diet; HMC: high microcystin content diet; HTHP: high temperature and high press treatment.

At the end of the experiment, the body weight gain rates of the fish fed with low toxic cyanobacterial bloom biomass (LMC) and the highly toxic cyanobacterial bloom treated with high temperature and high pressure (HTHP) were not significantly different from that of the control group, while the body weight gain rate of fish fed with high toxic cyanobacterial bloom biomass (HMC) was significantly lower than the control (between groups df = 3, within groups df = 8, F = 9.149, LMC: *p* = 0.226, HTHP: *p* = 0.255, HMC: *p* = 0.045) (Figure 1A). The feed efficiency of the fish fed the LMC and HTHP diets were not significantly different from the control, but the feed efficiency of the fish in the HMC treatment was significantly lower than the control after 54 days (between groups df = 3, within groups df = 8, F = 11.200, LMC: *p* = 0.376, HTHP: *p* = 0.288, HMC: *p* = 0.045) (Figure 1B). There were no significant differences in daily feed intake between the groups compared with the control(between groups df = 3, within groups df = 8, F = 1.086, LMC: *p* = 1.000, HTHP: *p* = 1.000, HMC: *p* = 1.000) after 54 days (Figure 1C), and there were no significant differences in alanine aminotransferase (ALT) or aspartate aminotransferase (AST) activity among the groups (Supplementary Tables S1 and S2) at the end of feeding trial (Figure 2).

### 2.2. Safety Evaluation of Tilapia Based on TDI in Different Treatments

**Figure 3:**
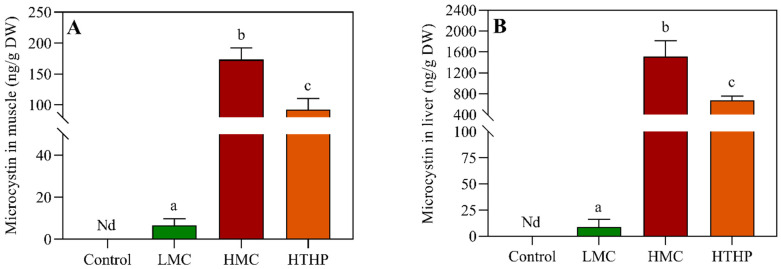
MC accumulation in tissues of tilapia. (**A**) Microcystin content in muscle under different treatments; (**B**) Microcystin content in liver under different treatments. Mean values with different superscripts are significantly different (*p* < 0.05). Nd: Not detected. Control: commercial diet; LMC: low microcystin content diet; HMC: high microcystin content diet; HTHP: high temperature and high press treatment.

Figure 3A,B shows the MC content in liver and muscle at the end of the trial. Accumulation of MC in muscle and liver increased with dietary MC. The MC levels in the muscle of LMC, HTHP, and HMC treatment groups were 6.6, 92.2, and 173.3 ng/g DW, respectively. The order of MC content in liver was HMC (1514.1) > HTHP (672.3) > LMC (8.9) ng/g DW. The MC content of the LMC treatment group was significantly lower than those of the HTHP and HMC groups(data of MC in muscle: between groups df = 3, within groups df = 17, F = 201.789, LMC and HTHP: *p* = 0.001, LMC and HMC: *p* = 0.000; data of MC in liver: between groups df = 3, within groups df = 18, F = 104.523, LMC and HTHP: *p* = 0.000, LMC and HMC: *p* = 0.000).

**Figure 4:**
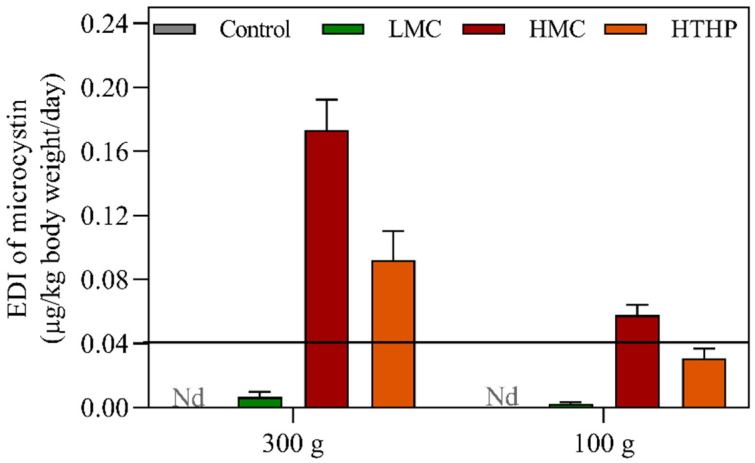
Estimated daily intake of MC by a person consuming 300 g or 100 g of muscle. The horizontal line indicates the WHO recommended maximum tolerable daily intake for humans (0.04 µg/kg body weight/day). Nd: Not detected. Control: commercial diet; LMC: low microcystin content diet; HMC: high microcystin content diet; HTHP: high temperature and high press treatment.

Compared to the TDI value of 0.04 µg/kg body weight/day [37], the EDI value was the estimated daily intake of 300 g or 100 g of fish muscle per day for an adult weighing 60 kg. If a person ate 300 g of fish muscle from the LMC (3.29 µg MC/g diet) treatment group per day, the daily intake of MC would be 0.006 µg/kg body weight/day, which is far below the limit of the TDI. If a person ingested 100 g of fish muscle from the LMC (3.29 µg MC/g diet) or HTHP (26.2 µg MC/g diet) treatment group per day, it also seems to be safe for human consumption (Figure 4).

### 2.3. Temporal Changes in MC Content from 2017 to 2019 in Lake Taihu

**Figure 5:**
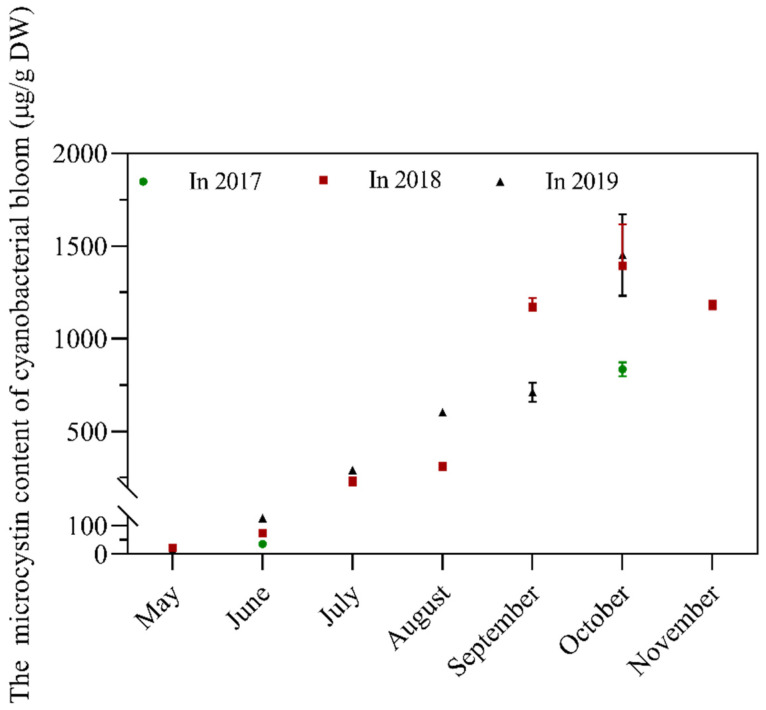
Temporal variation of MC content in cyanobacterial bloom biomass at N2 site, Meiliang Bay, Lake Taihu. The error bars suggest standard deviations (*n* = 3).

The cyanobacterial blooms at different stages of blooming were collected and freeze-dried for preservation, and the toxin content was analyzed. The MC content was always lower at the early blooming stage and was higher during the middle and late stages. The average MC content was 54 µg/g DW from May to June and 924 µg/g from August to October (Figure 5). The findings enabled us to obtain either low or high toxic cyanobacterial biomass from the same source, thereby facilitating the comparative study on the effects of low or high toxic *Microcystis* of biomass in diets on tilapia.

## 3. Discussion

In the present study, a 54-day growth experiment was conducted on tilapia to explore the effects of aquafeed containing *Microcystis* biomass on the growth performance and MC accumulation in the fish. The results showed that compared with the control group, there were no significant differences in the body weight gain rate or feed efficiency in LMC (3.29 µg MC/g diet) or HTHP group (26.2 µg MC/g diet). However, the body weight gain rate and feed efficiency of the HMC (35.3 µg MC/g diet) group were significantly lower than those of the controls. In addition, the toxin accumulation in the muscle tissue of the LMC experimental group was far below the TDI value recommended by the WHO, suggesting that the fish muscle seemed to be safe for human consumption. The findings demonstrated that it was feasible to add 18.5% of low toxic cyanobacterial biomass as a protein substitute in tilapia feed.

Several studies have been conducted to assess the feasibility of cyanobacterial bloom biomass as a protein substitute for tilapia [24,27,38]. Zhao et al. (2006a) reported that a diet containing *Microcystis* bloom (3.3 µg MC/g diet) had no negative effects on the feed conversion efficiency of the Nile tilapia, and the muscle was suggested to be safe for human consumption [24]. This result was similar to our finding that tilapia can grow well and be safe to consume when the toxin level is below 3.29 µg/g. However, the difference between the two studies lies in the amount of cyanobacterial biomass in the feed: in Zhao’s experiment, the proportion of cyanobacterial biomass was 3.51%, while in our study this was 18.5%. It seems that the content of microcystin in cyanobacterial bloom may determine the proper percentage of addition. The higher the level of toxin, the lower the percentage addition of cyanobacterial biomass. Zikova et al. (2010) illustrated the impact of a diet containing microcystin on stress and physiological growth in tilapia [38]. They found that growth was not significantly affected, even when the proportion of cyanobacterial bloom in the diet was as high as 20% (19.54 µg MC/g diet). The authors therefore proposed that cyanobacterial bloom might be even used as a component in the fish diet for Nile tilapia. This again helps to establish the perception that cyanobacterial bloom with low toxicity can be added to aquafeed in a higher proportion. This perception is also supported by the finding that growth performance was affected in tilapia exposed to a diet with a “high” toxin content [27].

A popularly cultivated fish, tilapia has also been applied for the purpose of cyanobacterial bloom control due to its capacity to ingest and digest cyanobacteria [39,40,41]. The ability to eliminate cyanobacterial bloom via tilapia may be due to the species’ high depuration rate when feeding on fresh toxic cyanobacteria [42] or on diets containing toxic cyanobacteria [27]. Besides tilapia, the responses to diets containing cyanobacterial biomass have been evaluated in other fish species such as yellow catfish, gibel carp, hybrid sturgeon, and threadfin shad. Considering the similarity in experimental design and facilities, the results obtained from the same research group on yellow catfish [26], gibel carp [43], and hybrid sturgeon [28] were compared to evaluate those species’ tolerance to cyanobacterial bloom. The ability to tolerate cyanobacterial bloom biomass was hybrid sturgeon > yellow catfish > gibel carp. In the cases of threadfin shad [22] and Sacramento splittail [23], the studies emphasized the physiological and biochemical responses upon exposure to diets containing cyanobacterial bloom, including histopathological indicators, RNA/DNA ratio, and caspase activity. Those studies proposed that these parameters were more sensitive to toxin than growth parameters and therefore were probably indicative of the toxicity. ALT and AST are two of the most commonly used diagnostic biomarkers of liver disease and hepatocyte damage. Increased levels of these enzymes may be clinical features of microcystin exposure in medical and veterinary settings [44,45]. Previous studies in fish species such as common carp, silver carp, or goldfish also found that pure MC-LR (injected IP), lysates of cyanobacteria (applied per os) or exposure to cyanobacterial bloom in the field could cause significant changes in plasma enzyme activities [46,47,48]. However, plasma ALT and AST were not significantly increased when the tilapia were fed with LMC and HMC in the present study. These results indicate that the low toxin dietary *Microcystis* may slightly induce a liver stress in tilapia, yet the stress was not high enough to incite damage to the liver. In consideration of the reports that both medaka fish [49] and *Daphnia magna* [50] were adversely affected after exposure to living non-toxic *Microcystis* or its extracts, it is necessary to conduct the evaluation if the non-toxic or low-toxin-containing *Microcystis* biomass as a fish diet may exert a negative effect on fish upon chronic exposure. In addition, strict surveillance has to be applied during the cultivation of tilapia in fish ponds or in reservoirs where toxin may present and penetrate to farmed tilapia [51,52].

Although the response to a diet containing cyanobacterial bloom varied in different fishes, the limiting factors to the utilization of cyanobacterial bloom by fish are considered to be the toxin and the amount of cyanobacterial biomass added to the diet. The present study demonstrated that *Microcystis* bloom with low MC content could be added to aquatic feed in a relatively high proportion, suggesting that tilapia and even other fish could use cyanobacterial biomass with low toxin content. Thus, we consider our original question: is it possible to find a stable source of cyanobacterial biomass with low toxin content? The answer to this question is yes. Based on three years of data from this study, the MC average content in algal powder harvested from May to June was 54 µg/g DW with a minimum of 15 µg/g; in contrast, the average MC content from August to October was 924 µg/g DW. Importantly, the pattern of variation in MC of cyanobacterial bloom in Lake Taihu agreed with the results of previous studies in our laboratory. We previously demonstrated that the dominant species in the early blooming period was *M. flos-aquae*, a species that has generally been considered as a low or non-toxic species over the past decade [30,53]. Similar phenomena were also found in other lakes. For example, in Lake Dianchi, the percentages of toxic *Microcystis* were low from September through April [33]. In Lake Oneida, the proportion of toxic *Microcystis* was below the quantifiable limit in June [31]. On the other scenario, the change of toxicity of cyanobacterial bloom in lakes was also reported along with multi-year observation. In Lake Kinneret, the *Microcystis* community structure has shifted from high abundance of toxic species to less or non-toxic species multi-annually [54]. Altogether, we propose that it is not difficult to find out a stable source of cyanobacterial biomass with low toxicity or even non-toxic in many lakes.

Recently, harvesting cyanobacterial bloom biomass has been widely adopted as a major measure for cyanobacterial bloom control and mitigation in several large lakes in China that have suffered from heavy cyanobacterial bloom. In Lake Taihu, for example, over 10,000 tons of dry algal powder was obtained through a series of processing steps by the algae–water separation stations. It is estimated that about 20% of the dry biomass could potentially be utilized, since the toxin content was very low (about 20-fold less than that of toxic bloom). With the provision of low toxic bloom biomass, the utilization of cyanobacterial bloom as a substitute protein in the diet of fish will be facilitated, and this will generate practical applications in the near future.

## 4. Conclusions

Over three years of investigation, we found a stable source of low toxic cyanobacterial bloom in Lake Taihu. Due to the availability of low and high toxic cyanobacterial bloom from the same source, we demonstrated that it is feasible to add 18.5% low toxic cyanobacterial biomass as a protein substitute in tilapia feed. Since there is a constant source of low toxic cyanobacterial biomass, with further research and testing, the application of cyanobacterial bloom as a substitute protein in aquafeed among others can be advanced.

## 5. Materials and Methods

### 5.1. Tilapia, Cyanobacterial Biomass, and Experimental Diets

The Genetically Improved Farmed Tilapia used in the trial were obtained from Mingde Fish Hatching and Breeding Co., Ltd., Huanggang, China, and were acclimatized for two weeks. During the acclimation period, the fish were fed a commercial diet twice daily (9:00 and 15:00).

Cyanobacterial bloom samples were taken in Lake Taihu, Wuxi, China from 2017 to 2019. After sampling, fresh cyanobacteria were freeze-dried and stored at −20 °C. In the samples, *M. flos-aquae* was dominant in June, and *M. aeruginosa* dominated in October. Cyanobacterial bloom biomass used in the experiment was obtained from the samples collected in June and October 2017, and the MC content was about 0.04‰ and 0.8‰ of dry weight, respectively.

Four isonitrogenous and isocaloric trail diets were formulated (Table 1). The control diet used commercial feed without cyanobacterial biomass, while 18.5% of cyanobacterial bloom harvested in June were used to formulate the diet for a low microcystin content group (LMC), and 18.8% of cyanobacterial biomass collected in October were used to formulate the diet of a high microcystin content group (HMC). The HTHP group diet contained 18.8% cyanobacterial biomass that was gathered in October. Both HTHP and HMC treatments had the same source of *Microcystis* with higher toxin content. HTHP has further been processed with high temperature and high pressure (set as: screw speeds 200 rpm, barrel temperatures 150 °C), and its final toxin content was reduced during the process. All diets were made into small pellets using an extrusion machine, dried at 60 °C, and stored at −4 °C.

### 5.2. Experimental Procedure and Sample Collection

The growth experiment was carried out in an indoor recycling aquaculture system. At the beginning of the growth trial, the fish were starved for 24 h. Healthy and similar juvenile fish were selected (initial weight: 15.75 g) and randomly assigned to 12 fiberglass tanks (diameter 1.5 m, volume 300 L). Each tank contained 30 individuals. This experiment was conducted for 54 days, and the detailed conditions are shown in Table 2. At the end of the trial, fish were starved for 24 h before being collected, and all fish were weighed. Six fish were randomly selected from each treatment and dissected on an ice pan. The liver and muscle tissues of these fish were removed and frozen at −20 °C. The samples were freeze-dried and ground into powder for toxin determination.

### 5.3. Microcystin Analysis

Freeze-dried algal powder was extracted with 5% (*v*/*v*) acetic acid for 40 min by a magnetic stirrer, centrifuged at 7000 rpm for 10 min, and the supernatant was transferred to a new bottle. The residues were extracted with 80% (*v*/*v*) methanol solution for 1 h. After evaporation of the methanol, the extracts were mixed and passed through preconditioned Sep-Pak C18 cartridges. The cartridges were preconditioned with methanol and Milli-Q water (Millipore Co., Burlington, MA, USA).

The MC extraction process of fish tissue samples was the same as for the algal powder according to the above-mentioned extraction steps, and the residues were re-extracted with methanol [26]. Finally, MC was dissolved in solution with 1 mL of ultrapure water and stored at −20 °C for MC analysis. All samples were determined by the enzyme-linked immunosorbent assay (ELISA) (Institute of Hydrobiology, Chinese Academy of Sciences, Wuhan, China) for total free microcystin [55].

### 5.4. Risk Assessment

According to the tolerable daily intake (TDI) of 0.04 µg/kg body weight/day proposed by the WHO, the estimated daily intake (EDI) value was calculated and compared with the TDI for risk assessment [37]. The EDI value was the estimated daily intake of 300 g or 100 g of fish muscle per day for an adult weighing 60 kg.

EDI (µg/kg body weight/day) = MC (µg/g dry weight) × [300/5 (or 100/5)] (g dry weight)/60 (kg)

Where MC (µg/g dry weight) was the concentration of microcystin in the muscle tissue, 5 was a coefficient applied to convert wet weight to dry weight [26], and 300 g fish muscle in wet weight can be converted to 60 g dry weight by a coefficient 5.

### 5.5. Enzymatic Analysis

Plasma alanine aminotransferase (ALT) and aspartate aminotransferase (AST) were evaluated by an automatic biochemistry analyzer BS-460 manufactured by Mindray, using standard kits (ALT 105-000442-00, AST 105-000443-00) according to the manufacturer’s instructions (Mindray Bio-Medical Electronics Co., Ltd., Shenzhen, China).

### 5.6. Calculations and Statistical Analysis

Body weight gain rate (%) = 100 × [final body weight (wet weight, g) − initial body weight (wet weight, g)]/initial body weight (wet weight, g).

Feed efficiency (%) = 100 × [final body weight (wet weight, g) − initial body weight (wet weight, g)]/food intake (dry matter, g).

Daily feed intake (g DM/ind. /day) = food intake (dry matter, g)/average fish numbers/days.

### 5.7. Data Analysis

All data analyses were performed using one-way analysis of variance followed by Bonferroni tests (for homogeneity of variance) or Games–Howell (for heterogeneity of variance) (PASW Statistics 18). The natural log transformation was performed on ALT and AST parameters, in order to fulfill the normality requirement. Significant differences between treatment groups were considered at *p* < 0.05. The data were visualized using GraphPad Prism 8.0.2 (GraphPad Software Inc., La Jolla, CA, USA).

## Figures and Tables

**Table 1:** Formula and chemical composition of experiment diets (g/kg in dry matter).

Ingredients	Control	LMC	HMC	HTHP
Fish meal	330	220	220	220
Soybean meal (Oil-extracted)	120	80	80	80
Rapeseed meal	120	80	80	80
Blood-meal	20	20	20	20
Starch	190	210	210	210
LMC	0	185	0	0
HMC	0	0	188	0
HTHP	0	0	0	188
Yeast food attractant	10	10	10	10
Mineral premix ^a^	50	50	50	50
Vitamin premix ^b^	5	5	5	5
Fish oil	23	29	28.5	28.5
Soybean oil	23	29	28.5	28.5
Cellulose	109	82	80	80
Chemical composition				
Moisture	68	76	65	70
Crude protein	363	362	369	365
Crude lipid	85	85	83	84
Energy (MJ/Kg DM)	169	167	168	167
Microcystin content (µg/g DW)	0.00	3.29	35.3	26.2

^a^ Mineral premix (mg/kg diet, H440): NaCl, 500; MgSO_4_·7H_2_O, 7500; NaH_2_PO_4_·2H_2_O, 12,500; KH_2_PO_4_, 16,000; Ca(H_2_PO_4_)·2H_2_O, 10,000; FeSO_4_, 1250; C_6_H_10_CaO_6_·5H_2_O, 1750; ZnSO_4_·7H_2_O, 176.5; MnSO_4_·4H_2_O, 81; CuSO_4_·5H_2_O, 15.5; CoSO_4_·6H_2_O, 0.5; KI, 1.5; starch, 225. ^b^ Vitamin premix (mg/kg diet, NRC, 1993): Thiamin, 20; riboflavin, 20; pyridoxine, 20; cyanocobalamine, 2; folic acid, 5; calcium patotheniate, 50; inositol, 100; niacin, 100; biotin, 5; starch, 3226; vitamin A (ROVIMIXA-1000), 110; vitamin D3, 20; vitamin E, 100; vitamin K3, 10.

**Table 2:** Conditions about the details of the experiment.

Methods	Conditions
Replication	3 tanks
Density (tail/per tank)	30
Temperature	25–28 °C
Light period	8:00–20:00
Water-dissolved oxygen	>7.4 mg/L
Ammonia-N	<0.5 mg/L
Feeding practice	By hand to apparent satiation twice daily 9:00–10:00, 15:00–16:00

## Data Availability

Not applicable.

## Supplementary Materials

The following are available online at https://www.mdpi.com/article/10.3390/toxins13100735/s1, Table S1: The Bonferroni tests of ALT, Table S2: The Bonferroni tests of AST.
