# Supplementary material for: Evaluation of Cyanobacterial Bloom from Lake Taihu as a Protein Substitute in Fish Diet—A Case Study on Tilapia
Source: Toxins (Basel). 2021 Oct 19;13(10):735. doi: 10.3390/toxins13100735 (PMC8538822; doi:10.3390/toxins13100735)
Supplement: Supplementary file 1 [file toxins-13-00735-s001.zip › toxins-1365928-SI.pdf]

Supplementary Materials: Evaluation of Cyanobacterial Bloom from Lake Taihu as a Protein Substitute in Fish Diet—A Case Study on Tilapia

Yan Huo, Yuanze Li, Wei Guo, Jin Liu, Cuiping Yang, Lin Li, Haokun Liu and Lirong Song

All variables were checked for their normality using the SPSS. The natural log transformation was performed on ALT and AST parameters, in order to fulfill the normality requirement.

ANOVA analysis of variance of ALT between control and all treatments showed: between groups, df = 3; within groups, df = 20; F = 1.336, and $p = 0.291$, indicating homogeneity of variance. With the Bonferroni tests, the p value results are shown in the table S1.

In regard to AST, the same analysis between control and all treatments showed: between groups, df = 3; within groups, df = 19; F = 0.837, and $p = 0.490$, indicating homogeneity of variance. With the Bonferroni tests, the p value results are shown in the table S2.

Table S1. The Bonferroni tests of ALT.

Treatment (I)	Treatment (J)	Difference (I-J)	*p* value
Control	LMC	−0.6776506	0.396
	HMC	−0.4637355	1.000
	HTHP	−0.4485304	1.000
LMC	Control	0.6776506	0.396
	HMC	0.2139151	1.000
	HTHP	0.2291202	1.000
HMC	Control	0.4637355	1.000
	LMC	−0.2139151	1.000
	HTHP	0.0152051	1.000
HTHP	Control	0.4485304	1.000
	LMC	−0.2291202	1.000
	HMC	−0.0152051	1.000

TableS2. The Bonferroni tests of AST.

Treatment (I)	Treatment (J)	Difference (I-J)	*p* value
Control	LMC	−0.8150284	0.810
	HMC	−0.5193145	1.000
	HTHP	−0.4990686	1.000
LMC	Control	0.8150284	0.810
	HMC	0.2957140	1.000
	HTHP	0.3159599	1.000
HMC	Control	0.5193145	1.000
	LMC	−0.2957140	1.000
	HTHP	0.0202459	1.000
HTHP	Control	0.4990686	1.000
	LMC	−0.3159599	1.000
	HMC	−0.0202459	1.000
